# How does a biopsy of endoscopically normal terminal ileum contribute to the diagnosis? Which patients should undergo biopsy?

**DOI:** 10.3402/ljm.v9.23441

**Published:** 2014-02-19

**Authors:** Ali Riza Koksal, Salih Boga, Huseyin Alkim, Meltem Ergun, Mehmet Bayram, Damlanur Sakiz, Osman Ozdogan, Engin Altinkaya, Canan Alkim

**Affiliations:** 1 Department of Gastroenterology, Sisli Etfal Education and Research Hospital, Istanbul, Turkey; 2 Department of Pathology, Sisli Etfal Education and Research Hospital, Istanbul, Turkey

**Keywords:** Terminal ileum, ileoscopy, chronic ileitis, inflammatory bowel disease

## Abstract

**Background:**

Terminal ileum endoscopy and biopsy are the diagnostic tools of diseases attacking the ileum. However, abnormal histological findings can be found in endoscopically normal terminal ileum.

**Objective:**

This study was performed to evaluate the histopathological results of biopsies from endoscopically normal terminal ileum in order to determine pre-procedure clinical and laboratory factors predicting abnormal histopathological results, if any.

**Methods:**

A total of 297 patients who underwent colonoscopy and terminal ileum biopsy and had normal terminal ileum or a few aphthous ulcers in the terminal ileum together with completely normal colon mucosa were included in the study. The patients were grouped into two arms as normal cases and cases with aphthous ulcers. Histopathological and pre-procedural laboratory results of patients were analyzed according to their indications.

**Results:**

The terminal ileum was endoscopically normal in 200 patients, and 97 patients had aphthous ulcers. Chronic ileitis rate was present in 5.5% of those with endoscopically normal terminal ileum and in 39.2% of the patients with aphthous ulcers. In both groups, the highest rate of chronic ileitis was detected in the patients with known inflammatory bowel disease (IBD) (15.4 and 50%, respectively), anemia (9.5 and 43.5%, respectively), and in the patients having chronic diarrhea together with abdominal pain (7.7 and 44.8%, respectively). We found that the sensitivity of mean platelet volume for predicting chronic ileitis was 87% and the specificity was 45% at a cut-off value lower than 9.35 fl.

**Conclusion:**

In anemia indication or chronic diarrhea together with abdominal pain, the frequency of aphthous ulcers detected by ileoscopy and the frequency of chronic ileitis detected histopathologically despite a normal-appearing ileum were elevated.

The terminal ileum is the most distal segment of the small intestine and hosts many toxic substances, including bacteria, viruses, parasites, and digested food. Therefore, it is lined by a specialized lymphoid tissue of the immune system. Increases in lymphocytes, macrophages, and mast cells in response to luminal antigens are observed physiologically (1, 2). Diseases involving the terminal ileum may be classified as the inflammatory bowel diseases (IBD), infectious and parasitic diseases, and more rarely, neoplasia. In cases of suspected IBD, terminal ileum endoscopy and biopsy represent the gold standard in the differential diagnosis of the infectious, inflammatory, and non-inflammatory disorders that mimic IBD in symptoms and findings. In addition, conducting a terminal ileum biopsy during colonoscopy is a significant criterion that is indicative of completion of the colonoscopy. However, biopsies may sometimes fail in establishing a diagnosis. On the other hand, biopsy-associated hemorrhages and perforations, and variant prion infections that can colonize the terminal ileum due to its lymphoid dominant structure, such as the Creutzfeldt-Jakob disease that is resistant to sterilization of the endoscopic forceps, have been reported (3). Due to all these reasons, performing a biopsy of an endoscopically normal terminal ileum is controversial (3–10). Previous studies had reported rates of abnormal histological findings in 0.6 − 5.2% of biopsies taken from endoscopically normal terminal ilea (3–6, 10–14) . Among the literature studies assessing the normal-appearing terminal ileum biopsy results, no study could be detected that evaluated the procedure indication, pre-procedure laboratory data, and the number of samples collected at the same time. We performed this study to compare the histopathological results of ileal biopsies taken from endoscopically normal-appearing terminal ilea based on the procedure indication and the pre-procedure laboratory data, thereby determining the factors affecting the diagnostic value of the biopsies from the normal-appearing terminal ilea. In brief, we organized this study to answer the question ‘In which patients would it be beneficial to obtain a biopsy of the terminal ileum even if it appeared normal?’.

## Methods

A total of 297 patients between 18 and 65 years of age who presented to the Sisli Etfal Education and Research Hospital, Gastroenterology Endoscopy Unit, between January 2009 and December 2011 and found to have a completely normal colon mucosa and a normal ileal mucosa or less than five aphthous ulcers in the terminal ileum were included in the study. Colonoscopy procedures were performed by the same gastroenterologist using an Olympus colonoscope (Olympus GIF-180, Tokyo, Japan). The patients’ demographics, procedure indications, colonoscopy reports, ileal biopsy sample numbers, and the ileal biopsy pathology results were all recorded. Pathology preparations were re-assessed by an experienced pathologist. Complete blood count, C-reactive protein (CRP), erythrocyte sedimentation rate (ESR), ferritin, albumin, vitamin B12, and stool test results were retrospectively collected by accessing the laboratory records obtained not more than 2 weeks before the procedure.

Two hundred and ninety seven patients were endoscopically grouped into two groups. Demographic data, procedure indications, pathologic findings, and laboratory data were compared between these two groups.

**Fig. 1:**
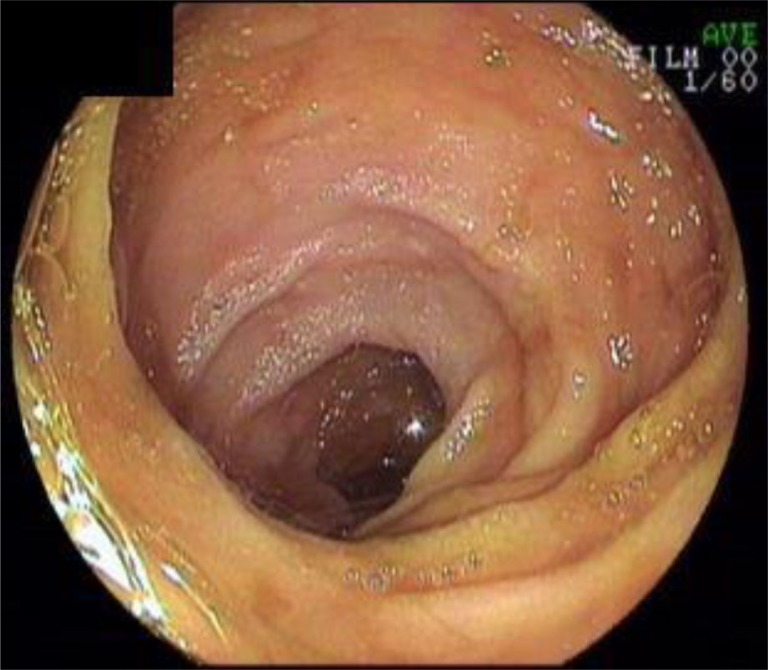
Endoscopic appearance of normal terminal ileum.

**Fig. 2:**
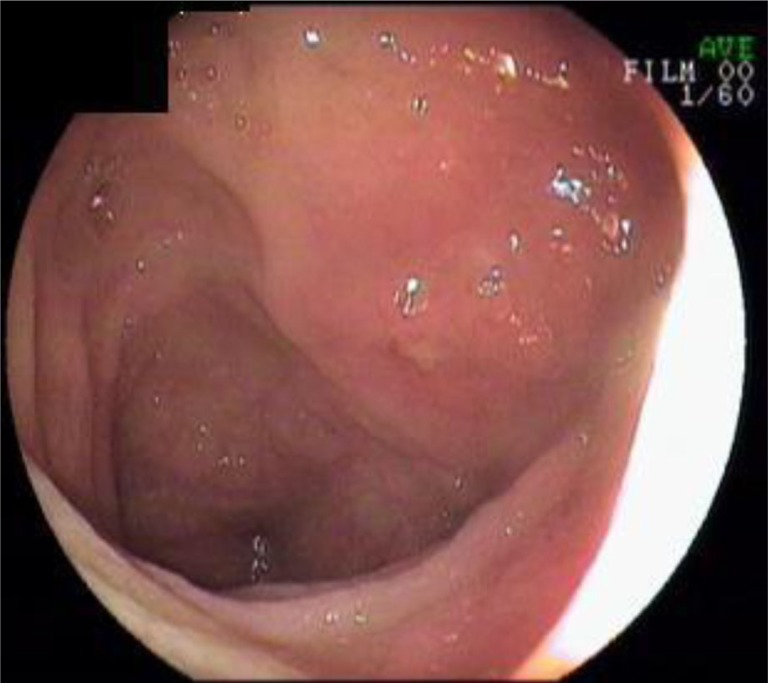
Aphthous ulcers on terminal ileum.

1. Patients with normal-appearing terminal ileum: Terminal ileum biopsies from patients with a normal colon and with no ulcer or erosion in the terminal ileum (*n*=200) (Fig. 1).
2. Patients with aphthous ulcer: Patients with a normal colon and less than 5 aphthous ulcers on a normal mucosa in the terminal ileum (*n*=97) (Fig. 2).

Patients were evaluated due to their indication for colonoscopy: anemia, chronic diarrhea, abdominal pain, chronic diarrhea with abdominal pain, presence of radiologic findings (wall thickening in the terminal ileum), and known IBD; there was also a control group (Table 1). Indications including cases of polyp surveillance, chronic constipation, and hematochezia were included in the control group.

**Table 1:** Distribution of patients according to the indication and frequency of terminal ileitis

Indication	Total investigated, *n* (%)	Chronic ileitis, *n* (%)
Anemia	44 (14.8)	12 (27.3)*
Chronic diarrhea	103 (34.7)	10 (9.7)
Chronic diarrhea+ abdominal pain	55 (18.5)	15 (27.3)*
Abdominal pain	22 (7.4)	1 (4.5)
Radiologic finding	18 (6.0)	2 (11.1)
Control	32 (10.8)	2 (6.3)
Known IBD	23 (7.8)	7 (30.4)*
Total	297 (100.0)	49 (16.5)

Histopathological chronic ileitis was more frequent in anemia, chronic diarrhea + abdominal pain, and known IBD groups

* (*p*<0.01). IBD, inflammatory bowel disease.

Histopathological findings were classified as normal, lymphoid hyperplasia, chronic ileitis and others. Cases with preserved villus and crypt structures; intact arteriole, venule, and lymphatic capillary structures; and normal number and distribution of cells were included in the normal group. The others group included cases with non-IBD diseases such as increased neutrophil count in the lamina propria, necrotizing granulomatous inflammation, and non-specific findings. The presence of distorted villous architecture (such as enlargement, blunting, broadening, atrophy, and irregular or diffuse shortening), branching, shortened or atrophic crypts, mixed inflammatory infiltrates in the lamina propria clearly distinct from the lymphoid follicles, and neutrophils infiltrating the epithelium were considered chronic ileitis.

Cases with more than five aphthous ulcers, deep ulcers, strictures or cobblestone finding at the terminal ileum, and with marked hyperemia, edema, fragility, and ulceration at colonoscopy were not included. Patients with malignancy, chronic renal failure, and liver failure, as well as patients without pre-procedure laboratory results were excluded from the study.

## Statistical methods

Data were analyzed using SPSS version 15 software. Pearson *Chi*-square test and Student's *t*-test were used for comparison of the categorical data and numeric data, respectively. The correlation analysis was demonstrated by Pearson and Spearman test while the independent efficacies of the parameters were demonstrated by linear regression analysis. Receiver operating characteristic curve (ROC) analysis was performed for the parameters that were found to be significant. All the statistical assessments were made at the 95% confidence interval; *p*<0.05 was considered to be statistically significant.

## Results

### General analyses

The mean age of the study group was 42±13.9 years. A total of 297 patients meeting the pre-specified criteria who underwent terminal ileum biopsy, including 142 females and 155 males, were included in the study. A hundred and three patients (34.7%) underwent colonoscopy due to chronic diarrhea and this was the most common indication in the whole study population (Table 1). The other frequent indications were chronic diarrhea together with abdominal pain (19%) and anemia (15%).

The histopathological evaluation detected chronic ileitis in 49 (16.5%) of the 297 patients. The diagnosis of chronic ileitis was found to be statistically significant more frequently in the indications of anemia, known IBD, and chronic diarrhea together with abdominal pain compared to all other indications (*p*<0.01).

### Group analysis for endoscopically normal terminal ileum

Two hundred (67%) of the patients had endoscopically normal terminal ileum and 97 (33%) of the patients had aphthous ulcer in the terminal ileum. There was no significant difference between the two groups with respect to age and gender.

The most common colonoscopy indication in the endoscopically normal terminal ileum group was also chronic diarrhea. Eighty-five of 200 patients (42.5%) underwent colonoscopy because of chronic diarrhea. Twenty-six (13%) patients underwent colonoscopy due to chronic diarrhea together with abdominal pain, 21 (10.5%) for anemia, 17 (8.5%) for abdominal pain, 13 (6.5%) for positive radiologic finding, and 13 (6.5%) for known IBD. Twenty-five (12.5%) patients who had colonoscopy for other reasons, such as constipation, meteorism, or colon cancer screening, were used as control.

**Fig. 3:**
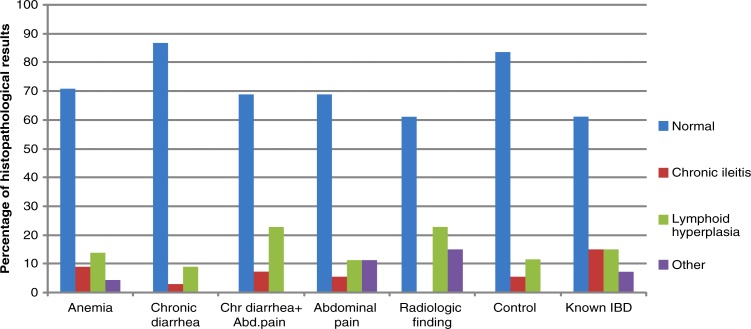
Histopathological results of biopsies from endoscopically normal terminal ileum according to indication.

The histopathological assessment of biopsies of endoscopically normal terminal ileum patients revealed normal findings in 156 of the 200 patients (78%), while 27 patients (13.5%) had findings consistent with lymphoid hyperplasia. Chronic ileitis was determined in 11 (5.5%) patients. Chronic ileitis was significantly more common in the groups of known IBD (15.4%), anemia (9.5%), and chronic diarrhea together with abdominal pain (7.7%) than in the other groups (*p*<0.05) (Table 2, Fig. 3). Two of the chronic ileitis patients were known IBD patients. Six (66%) of the remaining nine patients were diagnosed with Crohn's disease (CD) after further evaluation or during follow-up. One patient was lost from follow-up and the last two patients remained undiagnosed.

**Table 2:** Pathology results of biopsies from endoscopically normal terminal ileum according to indication

		Pathology							
		
		Normal		Lymphoid hyperplasia		Other		Chronic ileitis	
					
Indication	*n*	*n*	%	*n*	%	*n*	%	*n*	%
Anemia	21	15	71.4	3	14.3	1	4.8	2	9.5*
Chronic diarrhea	85	74	87.1	8	9.4	0	0.0	3	3.5
Chronic diarrhea + abdominal pain	26	18	69.2	6	23.1	0	0.0	2	7.7*
Abdominal pain	17	12	70.6	2	11.8	2	11.8	1	5.9
Radiologic finding	13	8	61.5	3	23.1	2	15.4	0	0.0
Control	25	21	84.0	3	12.0	0	0.0	1	4.0
Known IBD	13	8	61.5	2	15.4	1	7.7	2	15.4*
Total	200	156	78.0	27	13.5	6	3.0	11	5.5

Chronic ileitis was more frequent in anemia, chronic diarrhea + abdominal pain, and known IBD groups

* (*p*<0.05). IBD, inflammatory bowel disease.

In patients diagnosed with chronic ileitis, the mean number of samples examined was 4.8 (min: 1, max: 11) and in those considered normal it was 2.3 (min: 1, max: 5). This difference was statistically significant (*p*<0.01).

### Aphthous ulcer group analyses

**Fig. 4:**
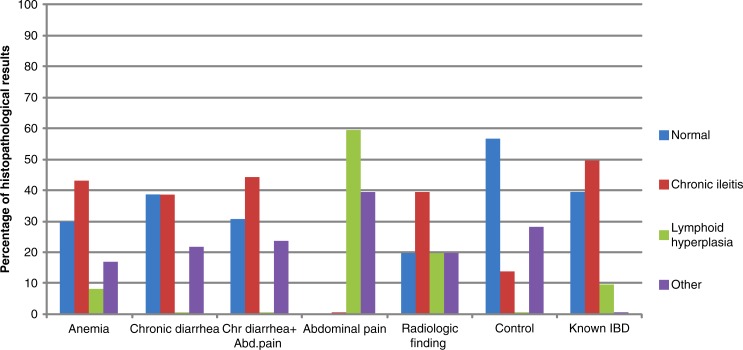
Histopathological results of biopsies from aphthous ulcer group according to indication.

While 11 of the 200 patients (5.5%) with a normal terminal ileum were diagnosed with chronic ileitis, 38 of the 97 patients (39.2%) with aphthous ulcer were found to have chronic ileitis. Chronic ileitis rate was significantly higher in the aphthous ulcer group relative to the endoscopically normal group (*p*<0.01). In the aphthous ulcer group, none of the abdominal pain cases and 14% of the control cases had chronic ileitis. In all of the remaining groups, chronic ileitis rate was 40% on average (Table 3, Fig. 4). Five of the 38 chronic ileitis patients were known IBD patients. Twenty-five (75%) of the remaining patients were diagnosed with CD after further evaluation or during follow-up. Two patients were lost from follow-up and the other six patients remained undiagnosed.

**Table 3:** Pathology results of biopsies from terminal ileum with aphthous ulcer according to indication

		Pathology							
		
		Normal		Lymphoid hyperplasia		Other		Chronic ileitis	
					
Indication	*n*	*n*	%	*n*	%	*n*	%	*n*	%
Anemia	23	7	30.4	2	8.7	4	17.4	10	43.5
Chronic diarrhea	18	7	38.9	0	0.0	4	22.2	7	38.9
Chronic diarrhea + abdominal pain	29	9	31.0	0	0.0	7	24.1	13	44.8
Abdominal pain	5	0	0.0	3	60.0	2	40.0	0	0.0
Radiologic finding	5	1	20.0	1	20.0	1	20.0	2	40.0
Control	7	4	57.1	0	0.0	2	28.6	1	14.3
Known IBD	10	4	40.0	1	10.0	0	0.0	5	50.0
Total	97	32	33.0	7	7.2	20	20.6	38	39.2

### Laboratory analyses

When we analyzed the pre-procedure laboratory data, we found that the white blood cell (WBC) and platelet counts were statistically significantly higher in the aphthous ulcer group in comparison to the endoscopically normal group (*p*<0.01). Also, acute phase reactants ESR and CRP levels were significantly higher and albumin and vitamin B12 levels were significantly lower in the aphthous ulcer group (*p*<0.05) (Table 4).

**Table 4:** Comparison of the laboratory data according to the endoscopic finding

	Normal terminal ileum, *n*=200	Aphthous ulcer, *n*=97	
		
	Mean±*SD*	Mean±*SD*	*p*
WBC	7,096±2,287	8,430±2,933	<0.001
Hematocrit	39.4±6.2	39±5.4	0.65
Platelet	259,140±87,424	299,048±105,937	0.002
MCV	82.5±8.3	83.5±6.8	0.35
MPV	10.1±1.01	9.9±0.7	0.07
CRP	10.3±22.6	19.9±31.8	0.015
ESR	17.9±22.9	27.04±18.8	0.016
Albumin	4.4±0.4	4.1±0.5	0.01
Ferritin	76.2±205.4	99.7±252	0.53
Vitamin B12	317±228	243.2±105.6	0.03

CRP, C-reactive protein; ESR, erythrocyte sedimentation rate; MCV, mean corpuscular volume; MPV, mean platelet volume; SD, standard deviation; WBC, white blood cell count.

Also, we compared pre-procedure laboratory data of patients having histopathological chronic ileitis with histopathologically normal patients (*n*=188). While doing this, we excluded patients with lymphoid hyperplasia (*n*=34) or other pathological diagnosis (*n*=26). WBC and platelet counts were statistically significantly higher in the chronic ileitis group compared with the normal group (*p*<0.01). Mean platelet volume (MPV) was found to be significantly lower in the chronic ileitis group (*p*<0.01). In addition, the mean ESR was higher in patients with chronic ileitis (*p*<0.05) (Table 5).

**Table 5:** Comparison of laboratory data according to the histopathological results

	Normal histopathology, *n*=188	Chronic ileitis, *n*=49	
		
	Mean±SD	Mean±SD	*p*
WBC	7,312±2,507	8,452±3,068	0.012
Hematocrit	39.8±6.2	39±5.8	0.470
Platelet	260,341±86,777	321,977±117,705	<0.001
MCV	82.3±8.8	82.9±5.4	0.550
MPV	10.2±0.8	9.4±0.6	<0.001
CRP	9.3±19.87	17.2±27.0	0.057
ESR	16±19.9	28.6±31.2	0.020
Albumin	4.4±0.4	4.1±0.6	0.051
Ferritin	74.2±214	59.9±78.7	0.730
Vitamin B12	316.2±225.8	246.1±163.8	0.100

CRP, C-reactive protein; ESR, erythrocyte sedimentation rate; MCV, mean corpuscular volume; MPV, mean platelet volume; SD, standard deviation; WBC, white blood cell count.

Linear regression analysis revealed two markers that can predict the histopathological diagnosis independently from the other parameters: MPV and ESR. The ROC analysis performed for these two parameters detected that for ileitis diagnosis with a cut-off value below 9.35 fl for MPV, the sensitivity was 87% and the specificity was 45% (Area under the curve (AUC): 0.76; *p*<0.01). For ESR, the sensitivity was 48% and the specificity was 43% at a cut-off value above 10.5 mm/h (AUC: 0.41; *p*=0.08).

## Discussion

Terminal ileum biopsies have a fundamental role in the diagnosis of IBD, particularly in CD. In cases of CD, the terminal ileum usually exhibits aphthous or linear ulcerations and cobblestone appearance. In addition, although the endoscopic appearance is almost normal in certain cases, biopsies may show histopathological changes that are consistent with chronic ileitis. The predominant opinion in the literature is that biopsies of terminal ileum with a normal-appearing mucosa during endoscopy have a low diagnostic value (3–6). In these studies, no ileal biopsy is recommended unless lesions are observed endoscopically. However, in several studies, even if the endoscopic appearance is normal, biopsy is recommended for patients with chronic diarrhea and/or abdominal pain. There have been cases of CD, intestinal tuberculosis, Cytomegalovirus (CMV) colitis, and microsporidiosis diagnosed by biopsy of the normal-appearing terminal ileum (7–10).

In the study by Melton et al. (5) on the diagnostic value of terminal ileum biopsies, the association between the clinical indication, endoscopic findings, and histopathological findings was evaluated. Based on retrospective data from approximately 10,000 patients, 52.2% of the patients underwent the procedure due to chronic diarrhea. By the procedure indication, the rate of abnormal ileal histopathology was found to be highest in the known or suspected CD group (36.4%). The most common indication was chronic diarrhea in our study as well (34%). Also, when compared according to procedure indication, the histopathological diagnosis of chronic ileitis was most commonly detected in the ‘known IBD’ (30.4%), anemia (27.3%), and ‘abdominal pain together with chronic diarrhea’ (27.3%) groups. In the study of Melton et al., 75% of the patients had an endoscopically normal-appearing terminal ileum, and among these patients, the rate of abnormal ileal histopathology was reported to be 5%. McHugh et al. (4) assessed the terminal ileum biopsy results of 414 consecutive patients with endoscopically normal terminal ileum and they found abnormal histopathology in 5.1% of patients. Similarly, 67% of our cases had a normal terminal ileum endoscopically and 5.5% of those with endoscopically normal terminal ileum had histopathological results consistent with chronic ileitis.

Sayilir et al. (10) compared the biopsies from terminal ileum with a normal endoscopic view in the colonoscopies performed for watery diarrhea and other indications. Chronic ileitis was detected at a rate of 5.9% in ileal biopsies in the watery diarrhea group. In our study, this rate was 3.5% in cases with chronic diarrhea and a normal-appearing ileum. In our study, the rate of chronic ileitis increased to 7.7% in the presence of chronic diarrhea and abdominal pain. As a result, we detected a rate of 5% for finding chronic ileitis in the normal-appearing terminal ileum group, consistent with the literature. No correlation was found between the number of ileum samples and the pathological diagnosis in the study by Sayilir et al. In our study, there was a statistically significant positive correlation between the number of samples and the diagnosis of chronic ileitis. We found that taking four or more biopsies increased the chronic ileitis diagnosis rate. So, increasing the number of biopsies to at least four can be recommended according to our results.

In a study by Melo et al. (1) performed in 2009, biopsy results of 111 cases with a normal-appearing terminal ileum were assessed. The comparison of a group with chronic diarrhea and/or abdominal pain to a group of asymptomatic patients or patients with other symptoms showed that the possibility of having mild to moderate ileitis was 2.5-fold higher. In our study, the rate of chronic ileitis was similarly higher in patients with chronic diarrhea and abdominal pain compared to isolated abdominal pain or other non-specific symptom groups.

In cases investigated for anemia, though the endoscopic view was normal, the rate of histopathologically detected chronic ileitis was high in our study. Similarly, in the study of Melton et al. (5), the rate of chronic ileitis in the normal endoscopic appearance group was higher in the anemia indication when compared to the abdominal pain, chronic diarrhea, and abnormal radiologic finding indications (6.1 vs. 5.1%).

ESR, CRP, WBC, platelet count, and albumin are used in detecting IBD activity, assessing response to treatment, detecting complications and relapses, and as a supportive tool in the differential diagnosis of IBD (15, 16). In their study, Cabrera-Abreu et al. (16) evaluated 153 pediatric cases with ‘possible IBD.’ Final diagnoses were compared by hemoglobin, platelet count, ESR, CRP, and albumin values. The highest correlation was found for hemoglobin and platelet count. No correlation was detected for ESR, CRP, and albumin. In our study, there was a statistically significant but weak correlation between these five pre-procedural parameters and chronic ileitis (in other words IBD). In the aforementioned study (16), the sensitivity and specificity of ESR values>10 mm/h for IBD diagnosis was 82 and 78%, respectively. In our study, at a cut-off value above 10.5 mm/h, the sensitivity and specificity of ESR for chronic ileitis diagnosis was 48 and 43%, respectively. Because our patient population did not include cases of evident IBD but rather undiagnosed, suspected mild cases and known IBD patients in remission, the acute phase reactants may be providing less information. Although the rate of chronic ileitis was found to be high in those undergoing colonoscopy for an anemia indication, there was no significant correlation between the hematocrit values and chronic ileitis.

MPV is a parameter that has recently been shown to be associated with inflammation and thrombosis. There are also studies on Ulcerative colitis (UC) and CD, which evaluate the correlation of MPV with inflammation and activation (17–24). For example, in the study by Jaremo et al. (21), a total of 27 patients including 18 UC and 9 CD cases were compared to the healthy control group with respect to MPV, and MPV was found to be statistically significantly lower in the IBD group (*p*<0.001).

Douda et al. (17) investigated the correlation of Crohn's disease activity index (CDAI) score, CRP, and MPV in 54 cases of CD and found that MPV significantly decreased in active disease. In a trial by Kapsoritakis et al. (22), where the active CD, inactive CD, and control groups were compared according to MPV, CRP, ESR, and WBC values, MPV was found to be negatively correlated to all the other inflammatory markers. The authors report that MPV could be a practical marker in demonstrating the activity. In our study, MPV shows a stronger and more independent correlation with the diagnosis of chronic ileitis relative to the other inflammatory markers, in line with all these studies. However, while the sensitivity (87%) of MPV for predicting chronic ileitis was high, the specificity (45%) was low. The data of our study indicate that a cut-off value of lower than 9.35 fl of MPV can be used as a determinative marker for the indication of terminal ileum entry and biopsy.

Based on the data from our study, the indication for requesting colonoscopy is important. The rate of detecting significant histopathological findings may be high in patients with abdominal pain together with chronic diarrhea and in patients with anemia even if the ileum appears normal. Terminal ileum intubation and biopsy increase the rate of diagnostic pathologic findings in these patient groups. As laboratory data, pre-procedure leukocyte, platelet, MPV, and ESR values may be used as a guide for entering the terminal ileum and taking a biopsy even if the mucosa appears normal.

In conclusion, in patients having anemia or chronic diarrhea together with abdominal pain whose MPV is lower than 9.35 fl and ESR higher than 10.5 mm/h, taking more than four biopsies from endoscopically normal terminal ilea will increase the diagnostic yield.
